# Bone morphogenetic protein dominantly suppresses epidermal growth factor-induced proliferative expansion of adult forebrain neural precursors

**DOI:** 10.3389/fnins.2015.00407

**Published:** 2015-10-29

**Authors:** Sandra E. Joppé, Laura K. Hamilton, Loic M. Cochard, Louis-Charles Levros, Anne Aumont, Fanie Barnabé-Heider, Karl J. L. Fernandes

**Affiliations:** ^1^Central Nervous System Research Group, Department of Pathology and Cell Biology, and Department of Neurosciences, Research Center of the University of Montreal Hospital, University of MontrealMontreal, QC, Canada; ^2^Department of Neuroscience, Karolinska InstitutetStockholm, Sweden

**Keywords:** adult neurogenesis, subventricular zone, BMP, EGF, neural precursors, mTOR, quiescence, proliferation

## Abstract

A single asymmetric division by an adult neural stem cell (NSC) ultimately generates dozens of differentiated progeny, a feat made possible by the proliferative expansion of transit-amplifying progenitor cells (TAPs). Although NSC activation and TAP expansion is determined by pro- and anti-proliferative signals found within the niche, remarkably little is known about how these cells integrate simultaneous conflicting signals. We investigated this question focusing on the subventricular zone (SVZ) niche of the adult murine forebrain. Using primary cultures of SVZ cells, we demonstrate that Epidermal Growth Factor (EGF) and Bone Morphogenetic Protein (BMP)-2 are particularly powerful pro- and anti-proliferative factors for SVZ-derived neural precursors. Dose-response experiments showed that when simultaneously exposed to both signals, BMP dominantly suppressed EGF-induced proliferation; moreover, this dominance extended to all parameters of neural precursor behavior tested, including inhibition of proliferation, modulation of cell cycle, promotion of differentiation, and increase of cell death. BMP's anti-proliferative effect did not involve inhibition of mTORC1 or ERK signaling, key mediators of EGF-induced proliferation, and had distinct stage-specific consequences, promoting TAP differentiation but NSC quiescence. In line with these *in vitro* data, *in vivo* experiments showed that exogenous BMP limits EGF-induced proliferation of TAPs while inhibition of BMP-SMAD signaling promotes activation of quiescent NSCs. These findings clarify the stage-specific effects of BMPs on SVZ neural precursors, and support a hierarchical model in which the anti-proliferative effects of BMP dominate over EGF proliferation signaling to constitutively drive TAP differentiation and NSC quiescence.

## Introduction

Neural stem cells (NSCs) are essential for the normal function, maintenance, and injury-induced repair of the adult central nervous system (CNS). Although only a small percentage of NSCs is cycling at any given moment, they are able to accomplish these critical functions by producing and maintaining a large pool of transit-amplifying progenitor cells (TAPs). In the forebrain subventricular zone (SVZ) niche, lineage analysis suggests that each TAP undergoes about 3–4 rounds of rapid proliferative expansion before differentiating, allowing a single asymmetric division by an activated NSC to ultimately give rise to as many as 32 neuroblasts (Ponti et al., 2013). TAP-derived neuroblasts and glial cells are required to maintain specific subpopulations of forebrain neural cells (Menn et al., 2006; Imayoshi et al., 2008; Luo et al., 2008) and to contribute to tissue repair following brain injury (Yamashita et al., 2006; Kolb et al., 2007; Benner et al., 2013). Since SVZ NSCs are typically quiescent, re-entering the cell cycle only once every 2–3 weeks (Morshead et al., 1994), NSC roles in CNS homeostasis and repair are critically dependent on both NSC activation and, importantly, the proliferative expansion of their downstream TAPs.

Proliferation of NSCs and TAPs (herein, collectively referred to as neural precursors) is promoted by many families of molecules within the SVZ niche (Gage, 2000; Alvarez-Buylla and Lim, 2004; Pathania et al., 2010; Bond et al., 2012), including the Epidermal Growth Factor (EGF) family. EGF family members signal through the Epidermal Growth Factor Receptor (EGFR), a cell surface tyrosine kinase receptor expressed in activated NSCs (Doetsch et al., 2002; Pastrana et al., 2009; Codega et al., 2014) and in the highly proliferative TAPs (Sun et al., 2005; Ferron et al., 2011). Ligand-induced activation of EGFR stimulates proliferation of activated NSCs and TAPs *in vivo* and allows neural precursor expansion as neurospheres *in vitro* (Reynolds and Weiss, 1992; Craig et al., 1996; Paliouras et al., 2012). Conversely, mice deficient for EGFR ligands show reduced SVZ neurogenesis (Tropepe et al., 1997). Multiple downstream signaling cascades are initiated upon EGFR activation, including the Akt-mTOR pathway. EGF treatment of cultured neural precursors stimulates mTOR activation within minutes, and EGF-induced mTOR activation is essential for proliferative expansion of the NSC lineage both *in vitro* and *in vivo* (Feliciano et al., 2011; Magri et al., 2011; Paliouras et al., 2012; Hartman et al., 2013).

The signaling mechanisms that counterbalance EGFR-induced proliferation in order to promote neural precursor quiescence are considerably less understood. Indeed, while many niche-derived factors modulate cell proliferation and/or neurogenesis within the SVZ (Pathania et al., 2010), how the ensemble of these signals is integrated within specific cell populations to coordinate the quiescence/proliferation/differentiation decisions of neural precursors is unclear. Here, we focused on bone morphogenetic proteins (BMPs). BMPs are expressed locally within the SVZ, where they have been shown to promote production of astrocytes at the expense of neurons and oligodendrocytes (Lim et al., 2000; Morell et al., 2014). Inhibition of endogenous BMP signaling modulates neurogenesis in both the SVZ and dentate gyrus niches, although there is conflicting data that likely reflects unresolved stage-specific effects on the neurogenic lineage and differences between niches (Lim et al., 2000; Bonaguidi et al., 2008; Colak et al., 2008; Gobeske et al., 2009; Mira et al., 2010; Guo et al., 2011; Bond et al., 2012, 2014).

In the present study, we ask how SVZ NSCs and TAPs integrate opposing pro- and anti-proliferation signals. Our findings reveal a hierarchical relationship between two key niche signals, EGFR ligands and BMPs, in regulating the quiescence/proliferation/differentiation decisions of SVZ neural precursors.

## Materials and methods

### Animals

C57BL/6 mice at 2–3 months of age were used for this study (Charles River). Experiments were conducted in accordance with the guidelines of the Canadian Council of Animal Care and were approved by the institutional ethics review committees of the University of Montreal and the Research Center of the University of Montreal Hospital (CRCHUM). *In vivo* procedures were performed with mice under isoflurane general anesthesia supplemented with injection of Buprivacaine local anesthetic (Hospira) (1 mg/kg). Mice were euthanized by injection of a mixture of ketamine (Bimeda-MTC)/xylazine (Bayer Healthcare)/acepromazine (Boehringer Ingelheim Canada Ltd) (200/10/2 mg/kg).

### Cell culture experiments

Clonally-derived neurosphere cultures were generated from the adult mouse striatum using a protocol based on Reynolds and Weiss (Reynolds and Weiss, 1992) and as detailed previously (Bouab et al., 2011; Paliouras et al., 2012). Standard neurosphere growth medium consisted of DMEM:F12 (3:1, Invitrogen), Penicillin/Streptomycin (1%, Wisent), Fungizone (1 mg/ml, Invitrogen), and B27 (2%, Invitrogen), supplemented with 20 ng/ml EGF (Sigma). In some experiments, we used additional extrinsic factors that included FGF-2 (Feldan), BMP-2 (Peprotech), PDGF-AA (Peprotech), VEGF-C (Peprotech), or SHH (Peprotech), as indicated. Cultures of primary neurospheres were passaged by mechanical dissociation and were expanded at a clonal plating density of 2000 cells/cm^2^. Primary neurospheres were used in quantitative neurosphere assays (i.e., counts of clonally-derived neurospheres). Secondary neurospheres were used when it was necessary to amplify neural precursor numbers for high-density adherent cultures for biochemical and flow cytometry analyses.

For the pro-proliferative assay, neurospheres were mechanically dissociated and plated at a high density of 25,000 cells/cm^2^ in a basal differentiation medium [i.e., neurosphere growth medium in which EGF was replaced with 2% Fetal Bovine Serum (FBS, Wisent)] and treated with 20 ng/ml of each extrinsic factor.

For the anti-proliferative assay, SVZ-derived cells were plated in non-adherent 24-well plates in medium containing a basal EGF concentration of 5 ng/ml (EGF^low^) to sustain neurosphere formation and treated on day 0 with 100 ng/ml of each extrinsic factor.

For the dose-response experiments, neurospheres were dissociated and plated on coated dishes at a density of 25,000 cells/cm^2^ for 6 days in medium containing 2% FBS to promote cell adherence and survival. Cells were treated on days 0 and 3 with the indicated concentrations of EGF and/or BMP-2.

For Olig2 labeling and Ki67-TUNEL double-labeling, neurospheres were dissociated and plated on coated dishes at a density of 25,000 cells/cm^2^ for 2 days in medium containing 5 ng/ml EGF (EGF^low^) and 1% FBS. Adherent cells were treated on day 0 with 100 ng/ml of EGF and/or BMP-2, and were processed after 2–3 days for immunofluorescence labeling.

For the experiments comparing the effects of transient treatment with BMP-2 and Rapamycin treatment, neurospheres were dissociated and plated at 25,000 cells/cm^2^ in medium containing 5 ng/ml EGF (EGF^low^) and 1% FBS, supplemented with either 100 ng/ml BMP-2 or 20 nM Rapamycin (Tocris). After 24 h of treatment (1 DIV), cells were washed twice with DMEM/F12 and then placed in medium containing 20 ng/ml EGF supplemented with 1% FBS for adherence/survival for an additional 4 days. Cells were lysed daily up to 5 DIV (i.e., up to 4 days after withdrawal of BMP-2 or Rapamycin).

For self-renewal assays, growth factor treatments were performed during primary neurosphere growth, then 60 average-sized primary neurospheres/condition were collected manually, mechanically dissociated, and the cells counted and re-seeded at clonal density (1.5 cells/μl) in standard neurosphere growth medium (above); this allowed quantification of the number of secondary neurospheres generated and retrospective calculation of the mean number of neurosphere-forming NSCs that were present per primary neurosphere.

Neurosphere numbers were quantified by plating cells in 24-well plates at the clonal densities indicated above (minimum of 8 wells/treatment/N). Neurosphere sizes were quantified by measuring the diameter of at least 100 neurospheres/condition using ImageJ software (version 1.47 v, NIH, USA), and the data expressed using frequency histograms [GraphPad Prism, Version 5.02 (GraphPad Software, Inc)].

### *In vivo* experiments

Intracerebroventricular (ICV) infusions were performed using 7 days osmotic pumps (Alzet, model 1007D, Durect) that were attached to brain infusion cannulae (Alzet, Brain infusion kit 3, Durect). Cannulae were stereotaxically implanted at coordinates: 0 mm anteroposterior (AP) and 0.9 mm mediolateral (ML) to the bregma. Growth factors were diluted in a vehicle solution (0.1% BSA in PBS) and infused at 400 ng/day. Animals were sacrificed after 5 days.

Adult brain electroporations were performed essentially as described previously (Barnabé-Heider et al., 2008). Briefly, plasmids were stereotaxically injected into the left lateral ventricle using a 1 ml Hamilton syringe (coordinates: 0 mm AP, 0.9 mm ML, 1.5 mm dorsoventral, relative to Bregma). Each animal received an ICV injection of 2 μl, delivered over 1.5–2 min, containing 10 μg of each plasmid (20 μg total). Electrical pulses (5 pulses, 50 ms intervals, 200 mv) were delivered using a square wave electroporator (ECM 830, Harvard Apparatus) with 7 mm Platinum Tweezertrodes (Harvard Apparatus). Animals were sacrificed after 3 days, which we determined to yield the peak number of plasmid-expressing cells.

Plasmids were amplified using Maxiprep (Promega), then the DNA precipitated and resuspended in 10 mM Tris-EDTA, pH 8, prior to use. GFAP::myrTomato and pGFAP::myrGFP were gifts from Robert Benezra (Addgene plasmids # 22671 and 22672 respectively) (Nam and Benezra, 2009). CS2 Flag-Smad6 was a gift from Joan Massague (Addgene plasmid # 14961) (Hata et al., 1998). Plasmids were precipitated with ethanol and resuspended in PBS prior to electroporation.

### Tissue processing

Anesthetized mice were perfused intracardially with 30 ml PBS pH 7.4 (Wisent) followed by 40 ml of 4% formaldehyde freshly prepared from paraformaldehyde (Acros). Brains were removed and post-fixed overnight at 4°C. Tissues were cut into 40 μm sections using a vibrating microtome (Leica VT1000S) and stored at −20°C in antifreeze solution (Bouab et al., 2011).

### Immunostaining and TUNEL

Immunostaining experiments were performed either using a 3,3′-Diaminobenzidine (DAB) detection step (brain sections) or a fluorescent secondary antibody detection step (brain sections and cell cultures), as described previously (Bouab et al., 2011; Grégoire et al., 2014). Antibodies are listed in Table 1.

**Table 1:** **Antibody list**.

**Antibody**	**Compagny**	**Western blot**	**Immunostaining**
Actin (mouse)	Abcam	1/20000	
Alexa-555 goat anti-mouse	Invitrogen		1/1000
Alexa-555 goat anti-guinea pig	Invitrogen		1/1000
Alexa-488 goat anti-chicken	Invitrogen		1/1000
Alexa-488 goat anti-rabbit	Invitrogen		1/1000
Alexa-647 goat anti-rabbit	Invitrogen		1/1000
CNPase (mouse)	Chemicon	1/500	
Doublecortin (guinea pig)	Chemicon		1/3000
Phospho-4EBP (Thr37/46) (rabbit)	Cell signaling	1/1000	
Phospho-p44/42 MAPK (ERK1/2) (thr202/tyr204) (rabbit)	Cell signaling	1/1000	
GFAP (chicken)	Novus biological		1/1000
GFAP (rabbit)	Dako diagnostic	1/1000	
HRP secondary Ab (anti-mouse)	Bio-rad	1/5000	
HRP secondary Ab (anti-rabbit)	Jackson immuno research	1/5000	
Ki67 (mouse)	BD biosciences		1/100
Olig2 (rabbit)	Chemicon	1/1000	1/250
Phospho-mTOR (Ser2448) (rabbit)	Cell signaling	1/1000	
Phospho-S6 (Ser240/244) (rabbit)	Cell signaling	1/1000	1/300
Sox2 (rabbit)	Chemicon	1/500	
Phospho-Smad1 (Ser463/465)/	Cell signaling	1/1000	
Smad5 (Ser463/465)/Smad8(Ser426/428) (rabbit)			
ßIII-tubulin (TUJ1) (mouse)	Covance	1/1000	

For Ki67-TUNEL double-labeling on cell cultures, immunostaining was first performed for Ki67 (1:100, mouse anti-human Ki67, BD Biosciences), and following the secondary antibody step, the TUNEL cell death assay (Roche) was used according to the manufacturer's instructions. Negative and positive controls were omission of dUTP and addition of DNAse (Sigma), respectively. Quantification of Ki67-TUNEL double-labeling was performed by counting the number of Hoechst-labeled nuclei that were double-labeled with either Ki67 or TUNEL from 20x photos (8 fields of view by condition).

Quantification of Ki67, Olig2, pS6, and DCX immunofluorescence staining was performed on brain sections following ICV infusions. Marker-positive cells were counted at 600x total magnification from 2 sections situated within 1 mm rostral to the level of infusion. Quantifications were performed contralateral to the side of pump implantation to avoid local injury responses, were restricted to the lateral (striatal) SVZ, and were normalized to the length of SVZ quantified (4 animals per group).

Quantification of GFAP::myrTomato or GFAP::myrGFP-expressing cells that co-expressed pS6 was performed following *in vivo* adult brain electroporations. Five to ten sections within the electroporated region of the SVZ were processed for pS6 immunostaining. GFAP::myrTomato or GFAP::myrGFP -expressing cells were then identified at 600x total magnification and assessed for co-expression of pS6. A total of 500–1400 reporter-expressing cells were assessed from a total of 7–8 animals per plasmid combination.

### Western blotting

Cultures analyzed by Western blotting were lysed in Ripa Buffer as previously described (Bouab et al., 2011). Protein samples for Western Blotting were prepared as described previously (Bouab et al., 2011; Paliouras et al., 2012), and primary antibody information is provided in Table 1. HRP-conjugated secondary antibodies were used at the following dilutions: anti-mouse IgG (1:5000, Bio Rad) or anti-rabbit IgG (1:5000, Jackson Immuno Research). Signals were revealed using the Clarity kit (Bio-Rad), detected using ChemiDoc (Bio-Rad), and quantified using Image Lab 4.1 software (Bio-Rad).

### Flow cytometry assays

#### Carboxyfluorescein diacetate Succinimidyl Ester (CFSE) cell division assay

To assess cell division, dissociated neurospheres were treated with 1 μM CSFE (Life technology) at 1 million cells/ml for 8 min at 37°C. The reaction was quenched with 100% FBS (1–2 min), and the labeled cells were then washed with PBS and DMEM/F-12 (3:1) medium and subsequently plated onto Poly-L-Lysine coated dishes at a density of 12,500 cells/cm^2^ in DMEM/F-12 (3:1) medium supplemented with 2% B27, 1% FBS, and 5 ng/ml EGF. Cells were treated on day 0 with 100 ng/ml of factors. After 3 days, the cells were harvested by trypsinization, washed and analyzed on an LSRII cytometer (BD Biosciences). Data analysis was performed using FlowJo v7.6.5 (Tree Star).

#### Propidium iodide and cell cycle analysis

Dissociated neurosphere cells were plated at 25,000 cells/cm^2^ in medium containing the indicated treatments. Cells were harvested by trypsinization after 24 h of treatment, rinsed and frozen in 70% ethanol in PBS until analysis. Prior to flow cytometry analysis, samples were treated with 0.5 mg/ml of DNAse-free RNAse (Sigma) (30 min at room temperature) and incubated with 50 mg/ml of propidium iodide (Sigma).

### Statistical analyses

Statistical comparisons were made using a One-Way analysis of variance (ANOVA) followed by either Dunnett's (comparison to a single control group) or Tukey's (multiple group comparison) *post-hoc* tests. For *in vitro* experiments, a repeated measures ANOVA was used as each animal had its own internal Vehicle control. All statistical analyses were performed using GraphPad Prism, Version 5.02 (GraphPad Software, Inc).

## Results

### EGF and BMP exert opposing effects on SVZ-derived neural precursor proliferation

In order to understand how SVZ neural precursors integrate conflicting signals, we first sought to identify two factors that exert opposing effects. To do so, we used the neurosphere culture system to perform pro- and anti-proliferative assays, and compared the effects of six distinct families of secreted niche factors.

**Figure 1:**
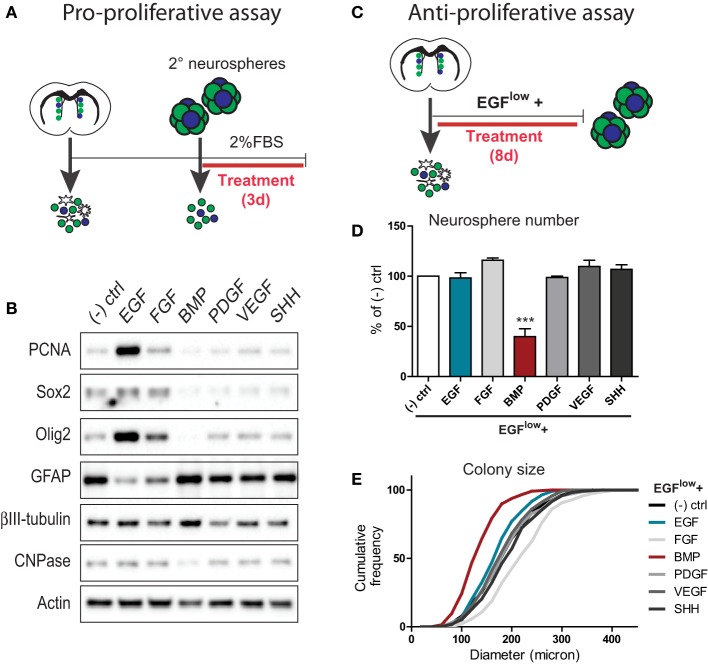
**Comparison of the pro- and anti- proliferative effects of 6 families of neurogenesis regulators on SVZ-derived neural precursor cultures. (A,B)** Assay for pro-proliferative effects. Experimental paradigm (see Materials and Methods for details) **(A)**. Western blotting analysis of cell lysates following growth factor treatments (representative example from 1 of 3 experiments) **(B)**. Note that EGF is the prominent stimulator of markers of proliferation (PCNA) and TAPs (Sox2, Olig2). **(C–E)** Assay for anti-proliferative effects. Experimental paradigm (see Materials and Methods for details) **(C)**. Quantification of the effects of growth factor treatments on neurosphere numbers **(D)** and sizes **(E)**. Note that BMP exerts a powerful anti-proliferative effect on neural precursors during neurosphere formation. ^***^p < 0.001, One-Way ANOVA (Dunnett's *post-hoc* test). Data are represented as mean ± SEM.

To identify proliferation-promoting SVZ signals, neurospheres expanded from the adult SVZ were dissociated and plated in a basal survival-promoting medium that was supplemented with 20 ng/ml of either EGF, FGF, BMP, PDGF, VEGF, or SHH (Figure 1A). Three days later, cells were lysed and analyzed biochemically for changes in proliferation and differentiation markers (Figure 1B). Western blot analysis showed that in comparison to the unsupplemented control, EGF and to a lesser extent FGF were the only factors that stimulated expression of proliferating cell nuclear antigen (PCNA) (Figure 1B). Consistent with this, EGF and FGF increased levels of Sox2 and Olig2, while decreasing levels of the astrocyte marker GFAP (the principal differentiated cell type in neurosphere-derived cultures) (Figure 1B). In this regard, Sox2 is expressed by both NSCs and TAPs in the SVZ. Although Olig2 normally identifies only a small subset of the SVZ TAP population, previous studies have shown that the vast majority of TAPs generated using exogenous EGF/FGF are Olig2+Sox2+ positive progenitors (Gabay et al., 2003; Lindberg et al., 2012). Thus, among these six factors, EGF is the prominent pro-proliferative factor for TAPs.

To identify anti-proliferative SVZ signals, neurospheres were grown from the dissociated SVZ using a low basal concentration of 5 ng/ml EGF (EGF^low^) to allow neurosphere formation and further supplemented with 100 ng/ml of each individual factor (Figure 1C). Quantifications showed that, at the clonal cell density used to grow neurospheres, treatment with 100 ng/ml EGF did not further increase the number (Figure 1D) or size (Figure 1E) of neurosphere colonies compared to the EGF^low^ control condition. Likewise, none of the other factors significantly increased neurosphere number over the EGF^low^ condition (Figure 1D), although FGF increased the mean colony size from 192.4 ± 3.3 to 227.2 ± 4.0 μm. Notably, however, BMP treatment reduced the number of neurospheres generated from the SVZ by more than 50%, and reduced the mean colony size to 139.1 ± 2.8 μm (Figures 1D,E).

Together, these assays identified EGF and BMP as particularly potent pro- and anti-proliferative factors for SVZ-derived neural precursors, respectively.

### TAPs: BMP suppresses EGF-induced proliferation via highly dominant effects on proliferation, differentiation, cell cycle parameters, and cell death

**Figure 2:**
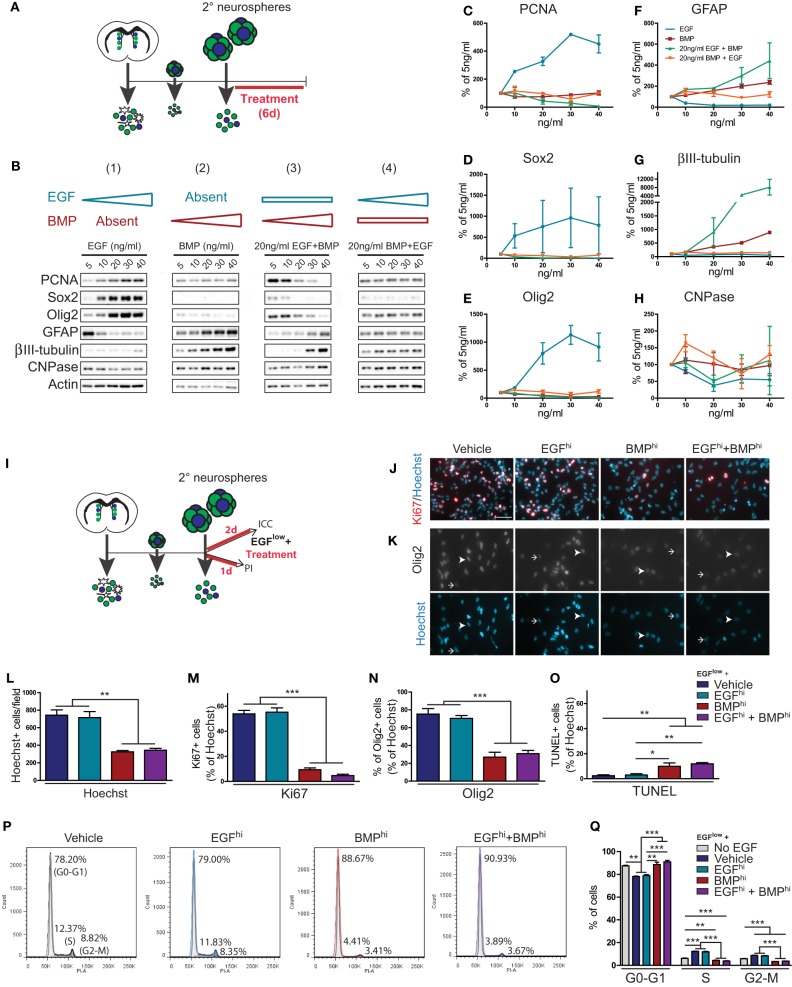
**BMP exerts dominant effects over TAP proliferation, differentiation, cell cycle parameters and cell death. (A–H)** EGF and BMP dose-response experiments. Experimental paradigm (see Materials and Methods for details) **(A)**. Western blot analysis of cell lysates following dose-response treatments (representative example from 1 of 2 experiments) **(B)**. Note that EGF-induced effects are suppressed in a dose-dependent manner by BMP, while BMP-induced effects are insensitive to EGF concentration. Densitometric quantification of markers of proliferation (PCNA) **(C)**, TAPs (Sox2, Olig2) **(D,E)** and neural subtypes (astrocytes, GFAP; neurons, ßIII-tubulin; oligodendrocytes, CNPase) **(F–H)**. **(I–Q)** Effect of EGF and BMP on cell proliferation, death and cell cycle. Experimental paradigms (see Materials and Methods for details) **(I)**. Immunofluorescence analysis for Ki67 **(J)** or Olig2 **(K)**, counterstained with Hoechst. Arrowheads and arrows show examples of positive and negative cells, respectively. Quantification of immunocytochemistry experiments showing the treatment effects on total number of Hoechst-positive nuclei **(L)** and proportion of cells that are positive for Ki67 **(M)**, Olig2 **(N)**, or TUNEL **(O)**. Cell cycle analysis by flow cytometry for DNA content using propidium iodide (representative example from 1 of 3 experiments) **(P)**, with quantification by cell cycle phase **(Q)**. Note that BMP has dominant effects on cell proliferation, cell cycle and cell death. Scale bar in **(J)** is 50 microns (for **J,K**). ^*^p < 0.05, ^**^p < 0.01, ^***^p < 0.001, One-Way ANOVA (Tukey *post-hoc* test). Data are represented as mean ± SEM.

TAPs constitute more than 95% of cells within individual neurospheres and express receptors for both EGF and BMP (Lim et al., 2000; Doetsch et al., 2002; Peretto et al., 2004; Pastrana et al., 2009; Codega et al., 2014). To examine how TAPs respond when simultaneously challenged with EGF and BMP, we used SVZ-derived neurospheres to generate high density adherent cultures for dose-response experiments. Adherent cultures were treated with either (1) increasing concentrations of EGF, (2) increasing concentrations of BMP, (3) intermediate levels of EGF plus increasing concentrations of BMP, or (4) intermediate levels of BMP plus increasing concentrations of EGF (Figures 2A,B).

Biochemical analysis showed that cells treated with increasing concentrations of EGF (5–40 ng/ml) exhibited a dose-dependent increase in markers of proliferation (PCNA) and TAPs (Sox2, Olig2) and suppression of astrocytic differentiation (GFAP). However, neurosphere-derived cells were rendered insensitive to these proliferation- and TAP-inducing effects of EGF when they were simultaneously exposed to 20 ng/ml BMP (Figures 2B–H). Conversely, BMP alone dose-dependently increased markers of differentiation and had no effect on proliferation and TAP markers; when simultaneously exposed to a constant 20 ng/ml EGF concentration, increasing the concentration of BMP suppressed EGF-induced TAP proliferation and increased differentiation in a dose-dependent manner (Figures 2B–H). Thus, these dose-response experiments showed that EGF and BMP have opposing effects on proliferation and differentiation of TAPs, and that the anti-proliferative/pro-differentiation effects of BMPs are highly dominant: when combined, BMP but not EGF is able to exert a dose-dependent suppression of the conflicting signal.

We next used immunocytochemistry and flow cytometry assays to better understand the cellular mechanisms involved in BMP's dominance over EGF (Figures 2I–Q). Secondary neurospheres were dissociated and plated for 48 h in a basal proliferation medium containing 5 ng/ml EGF (EGF^low^) that was further supplemented with either Vehicle, 100 ng/ml EGF (EGF^hi^), 100 ng/ml BMP (BMP^hi^), or both factors (EGF^hi^+BMP^hi^) (Figure 2I). EGF^hi^–treated cultures did not contain more cells than the Vehicle group, indicating that the basal EGF^low^ concentration maintained maximal proliferation over this time period. However, BMP^hi^ and EGF^hi^+BMP^hi^ groups both contained 55% fewer cells than the Vehicle- and EGF^hi^ groups (Figures 2J–L). Immunocytochemistry showed that BMP's effects are primarily anti-proliferative rather than pro-apoptotic. The proportion of cells that were in proliferation (Ki67+) decreased from 53.84 ± 2.80 and 55.19 ± 3.46% in the Vehicle and EGF^hi^–treated cultures to 9.34 ± 1.41 and 4.66 ± 1.11% in the BMP^hi^ and EGF^hi^+BMP^hi^ groups (Figures 2J–M). Similarly, Olig2 expression decreased from 75.19 ± 6.30 and 70.35 ± 3.34% in the Vehicle and EGF^hi^–treated cultures to 26.93 ± 5.50% and 30.86 ± 3.58% in the BMP^hi^ and EGF^hi^+BMP^hi^ groups (Figures 2K–N). There was also a smaller 7.5% increase in the proportion of cells that were apoptotic (TUNEL+) (Figure 2O). Consistent with BMP's anti-proliferative effects, propidium iodide (PI)-based flow cytometry analyses showed that the BMP^hi^ and EGF^hi^+BMP^hi^ conditions both shifted approximately 10% of neural precursors from the S- and G2-M phases of the cell cycle into G0-G1 within one day of treatment (Figures 2P,Q).

Notably, the effects of BMP^hi^ and EGF^hi^+BMP^hi^ conditions were virtually identical in all of these assays, revealing that the effects of BMP on neural precursor proliferation, cell cycle parameters, and apoptosis are all highly dominant to those of EGF.

### Distinct mechanisms of proliferation inhibition following EGF withdrawal and BMP treatment

**Figure 3:**
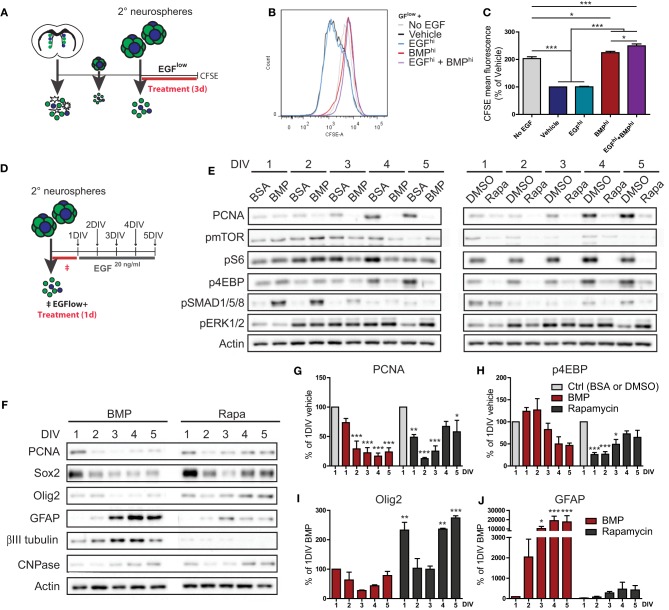
**Similarities and differences between BMP treatment and EGF withdrawal-induced suppression of TAP proliferation. (A–C)** Effect of EGF and BMP on proliferation kinetics. Experimental paradigm for CFSE experiment (see Materials and Methods for details) **(A)**. CFSE fluorescence intensity curves (representative example from 1 of 3 experiments) **(B)**, with mean fluorescence **(C)**. Note that BMP completely suppresses EGF-induced dilution of CFSE, a measure of the number of cell divisions, and is identical to EGF withdrawal. **(D–J)** Proliferation recovery assay following transient treatment with BMP or Rapamycin. Experimental paradigm (see Materials and Methods for details) **(D)**. Western blot analysis for proliferation (PCNA), mTORC1 pathway activation (pmTOR, pS6, p4EBP), SMAD pathway activation (pSMAD1/5/8), ERK pathway activation (pERK1/2), and actin loading control **(E)**. Representative example from 1 of 3 experiments. **(F–J)** Western blot analysis for PCNA, TAP (Sox2, Olig2), and differentiation (GFAP, ßIII-tubulin, CNPase) markers (representative example from 1 of 3 experiments) **(F)**. Densitometry quantifications **(G–J)**. Note BMP and Rapamycin both suppress proliferation, but BMP additionally stimulates differentiation and does not require a decrease in mTOR pathway activation. ^*^p < 0.05, ^**^p < 0.01, ^***^p < 0.001, One-Way ANOVA (Tukey *post-hoc* test in **(C)**, Dunnett's *post-hoc* test in **(G–J)**. Data are represented as mean ± SEM.

In order to explore the kinetics of BMP's suppression of EGF-induced proliferation, we performed a label retention-based assay using CarboxyFluorescein Succinimidyl Ester (CFSE). CFSE is a fluorescent dye that is passively incorporated into cells and then serially diluted from their cytoplasm during each subsequent cell division. Dissociated neurosphere cells were pre-labeled with CFSE, treated under various conditions for 3 days, and then analyzed for CFSE retention by flow cytometry (Figure 3A). As expected, control cultures plated under differentiation conditions of complete EGF withdrawal (No EGF) exhibited 2-fold more CFSE-fluorescence than the EGF^low^-containing Vehicle and EGF^hi^ conditions. Notably, BMP^hi^ and EGF^hi^+BMP^hi^-treated cultures closely resembled the No EGF differentiation condition, revealing that suppression of proliferation in response to BMP treatment follows similar kinetics as following EGF withdrawal (Figures 3B,C).

mTORC1 signaling is a key mediator of EGF-induced proliferative expansion of TAPs (Paliouras et al., 2012). Given the similar suppression of TAP proliferation between EGF withdrawal and BMP treatment conditions, we asked whether BMP operates by inhibiting mTORC1 signaling. Neurosphere-derived cells were exposed to either BMP or the mTORC1 inhibitor Rapamycin for 24 h, then switched to a proliferation medium containing 20 ng/ml EGF for up to 5 days to assess recovery of TAP proliferation and mTOR signaling (Figure 3D). Following treatment with the respective vehicle solutions (BSA for BMP or DMSO for Rapamycin), cells switched to the 20 ng/ml EGF proliferation medium exhibited a continual increase in expression of the proliferation marker PCNA accompanied by increasing expression of the TAP-associated mTORC1 pathway markers, pmTOR, pS6, and p4EBP (Figure 3E). In comparison, cells treated with either BMP or the reversible mTORC1 inhibitor Rapamycin displayed a rapid drop in PCNA levels (Figure 3E). In the case of Rapamycin, this drop was accompanied by immediate disappearance of the mTORC1 pathway markers, and PCNA and the p4EBP branch of the mTORC1 pathway subsequently recovered by day 4 (Figure 3E). In the case of BMP, the decrease in PCNA levels showed only a delayed and partial recovery, and notably, levels of pmTOR, pS6, and p4EBP did not parallel the reduction in PCNA, only displaying a slow and relatively minor decline over the 5-day period (Figure 3E). BMP-treated cells showed a strong and transient increase in pSMAD1/5/8, confirming BMP pathway activation (Figure 3E). Levels of pERK1/2 (a second proliferation-associated EGFR-induced pathway) were likewise relatively unchanged by BMP treatment (Figure 3E).

Further analysis confirmed that the recovery of proliferation following Rapamycin treatment was associated with a recovery of the TAP markers Sox2 and Olig2 (Figures 3F–J). In contrast, recovery of TAP markers following BMP treatment was limited, and instead, there was a strong increase in markers of differentiated astrocytes and neurons. The BMP differentiation response was primarily astrocytic, as immunocytochemistry showed the increase in βIII-tubulin was mainly due to enhanced neuronal morphological complexity (not shown).

This analysis reveals that despite the similarity in their time-course of proliferation inhibition, EGF withdrawal and BMP treatment are mechanistically distinct. EGF withdrawal, but not BMP treatment, is mediated by suppression of mTORC1 signaling. Moreover, TAPs transiently treated with Rapamycin undergo a reversible inhibition of proliferation, while TAPs transiently treated with BMP terminally differentiate into astrocytes despite the ongoing presence of EGF.

### NSCs: BMP promotes NSC quiescence by dominantly suppressing EGF-induced NSC activation

We next focused on the integration of EGF and BMP signals at the level of activated NSCs, which likewise express receptors for both EGF and BMP (Doetsch et al., 2002; Colak et al., 2008; Pastrana et al., 2009; Codega et al., 2014). To do so, we investigated the individual and combined impacts of these factors on growth and self-renewal of clonally derived neurospheres.

**Figure 4:**
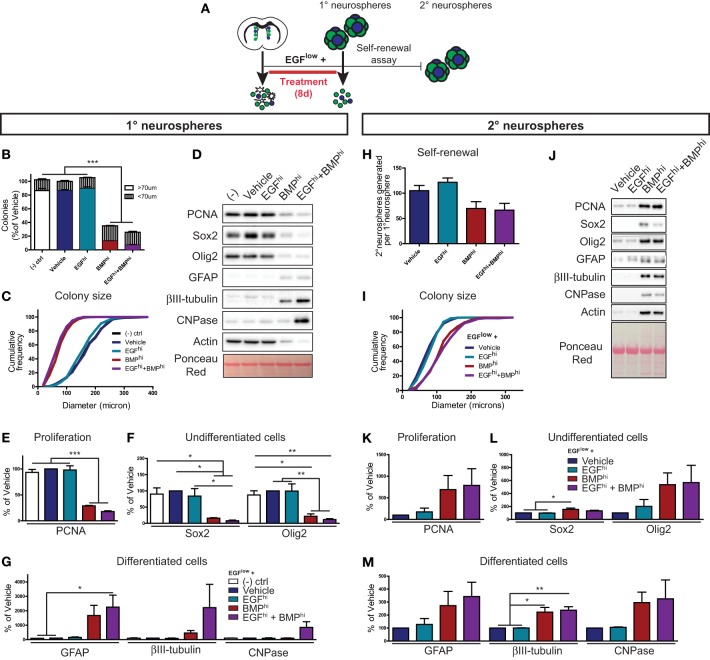
**BMP dominantly suppresses NSC activation. (A)** Experimental paradigm for neurosphere assay (primary neurospheres) and self-renewal assay (secondary neurospheres) (see Materials and Methods for details) **(A)**. **(B–G)** Primary neurosphere assay. Quantification of the number of floating colonies that are greater or less than 70 microns **(B)**. Cumulative frequency histogram of colony sizes **(C)**. Western blots of lysates from treated primary neurospheres (representative example from 1 of 3 experiments) **(D)**. Densitometric quantifications of markers of proliferation (PCNA) **(E)**, TAPs (Sox2, Olig2) **(F)**, and neural subtypes (astrocytes, GFAP; neurons, ßIII-tubulin; oligodendrocytes, CNPase) **(G)**. **(H–M)** Self-renewal assay (see Materials and Methods for details). Quantification of the average number of secondary neurospheres generated in normal proliferation medium from each primary neurosphere **(H)**. Cumulative frequency graph of colony sizes **(I)**. Western blots of lysates from secondary neurospheres (representative example from 1 of 3 experiments) **(J)**. Densitometric quantifications for markers of proliferation (PCNA) **(K)**, TAPs (Sox2, Olig2) **(L)** and neural subtypes (astrocytes, GFAP; neurons, ßIII-tubulin; oligodendrocytes, CNPase) **(M)**. Note that BMP-treated neurospheres remain capable of generating new neurospheres, and that neurospheres generated from NSCs that had been grown in the presence of BMP are larger with altered biochemical characteristics. ^*^p < 0.05, ^*^p < 0.05, ^***^p < 0.001, One-Way ANOVA (Tukey *post-hoc* test). Data are represented as mean ± SEM.

To determine whether EGF or BMP is dominant for activated NSCs, primary neurosphere colonies were grown at clonal density in the presence of 5 ng/ml EGF to sustain neurosphere formation (EGF^low^) and supplemented with either BSA (Vehicle), 100 ng/ml EGF (EGF^hi^), 100 ng/ml BMP (BMP^hi^), or 100 ng/ml EGF+100 ng/ml BMP (EGF^hi^+BMP^hi^) (Figure 4A). Quantification showed that there was no difference in the number (Figure 4B) or size distribution (Figure 4C) of neurosphere colonies between the Vehicle and EGF^hi^ conditions, confirming that the EGF^low^ concentration is saturating at the clonal cell density required in the neurosphere assay. Consistent with the anti-proliferative effect of BMP on TAPs described earlier, BMP^hi^ and EGF^hi^+BMP^hi^ conditions both significantly decreased neurosphere number and size (Figures 4B,C). Importantly, however, further analysis of the size distribution data showed that BMP reduced the number of full-sized colonies (larger than 70 μm) without increasing the number of small-sized colonies (less than 70 μm) (Figure 4B), indicating that BMP is also a negative regulator of neurosphere initiation (NSC activation). Biochemical analysis of these neurospheres showed that, as expected, EGF^low^ neurospheres that were Untreated, Vehicle- or EGF^hi^–treated all displayed high levels of the proliferation (PCNA) and TAP (Sox2, Olig2) markers and virtually undetectable levels of differentiation markers (βIII-tubulin, GFAP, and CNPase) (Figures 4D–G). However, this profile was reversed in the neurospheres obtained in the BMP^hi^ and EGF^hi^+BMP^hi^ conditions: BMP suppressed the expression of PCNA, Sox2, and Olig2, and induced expression of differentiated neuronal and glial markers (Figures 4D–G). Thus, in the colony-forming neurosphere assay, BMP overcomes the mitogenic effects of EGF at the level of both TAPs and NSCs.

To determine whether the BMP-induced decrease in neurosphere initiation was due to NSC *loss* (terminal differentiation, death, and/or irreversible senescence) vs. NSC *quiescence* (reversible slowing or exit from the cell cycle), we performed a neurosphere self-renewal assay. Specifically, we tested whether neurosphere-initiating NSCs are still present in neurospheres grown in the presence of BMP. Neurosphere colonies were grown in the same growth factor combinations as above, but following the generation of primary neurospheres, 60 neurospheres from each condition were dissociated and replated at clonal density in standard neurosphere-forming conditions (Figure 4A, “self-renewal assay”). Primary neurospheres grown in the BMP^hi^ and EGF^hi^+BMP^hi^ conditions again generated smaller primary neurospheres, and thus contained 65–70% fewer cells (data not shown) as compared to neurospheres grown in the Vehicle or EGF^hi^ conditions. However, when these smaller BMP-treated primary neurospheres were dissociated and replated under standard neurosphere-forming conditions, they were found to retain a disproportionately high number of neurosphere-initiating cells, exhibiting only a 30–35% decrease (not statistically significant) in secondary neurospheres generated (Figure 4H). Interestingly, further analysis of these secondary neurospheres showed that NSCs within the smaller BMP-treated primary neurospheres actually generated *larger* secondary neurospheres (Figure 4I). These larger secondary neurospheres exhibited a variety of biochemical differences, including enhanced expression of proliferation (PCNA), TAP (Sox2, Olig2) and differentiated (βIII-tubulin, GFAP, and CNPase) neural markers (Figures 4J–M).

These *in vitro* data reveal that (i) BMP is dominant over EGF at the level of activated NSCs, (ii) that BMP restrains NSC expansion by promoting a reversible quiescence rather than NSC loss, and (iii) that BMP-restrained NSCs subsequently exhibit an enhanced capacity for proliferation and differentiation.

### BMP limits EGF-induced proliferative expansion of TAPs *in vivo*

**Figure 5:**
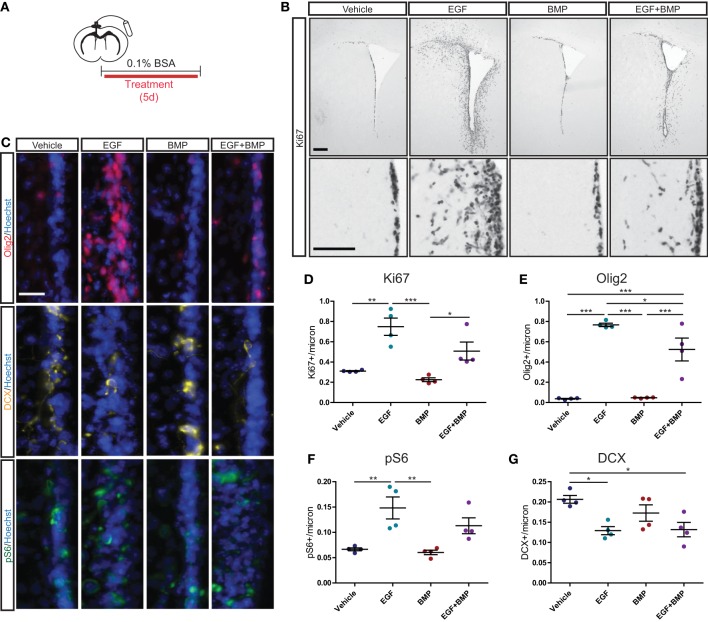
**BMP limits EGF-induced expansion of TAPs *in vivo***. Intracerebroventricular infusion paradigm (see Materials and Methods for details) **(A)**. Brightfield immunohistochemistry for Ki67, with low magnification (upper) and high magnification (lower) images of each condition **(B)**. Immunofluorescence for Olig2 (upper), DCX (middle) and pS6 (lower), each counterstained with Hoechst **(C)**. Quantification of Ki67+ **(D)**, Olig2+ **(E)**, pS6+ **(F)**, and DCX+ **(G)** SVZ cells (*N* = 4/group). Scale bars: **(B)** top: 250 microns, **(B)** bottom: 100 microns, **(C)**: 25 microns. ^*^p < 0.05, ^**^p < 0.01, ^***^p < 0.001, One-Way ANOVA (Tukey *post-hoc* test). Data are represented as mean ± SEM.

We next assessed whether the dominant anti-proliferative effects of BMP are also observed in an *in vivo* setting. Intracerebroventricular (ICV) infusions were used to deliver Vehicle solution (BSA), EGF, BMP, or a combination of EGF+BMP into the lateral ventricles of adult mice (Figure 5A). Immunohistochemical analysis after 5 days of infusion indicated that EGF dramatically increased Ki67+ cell proliferation surrounding the lateral ventricles, that BMP had no detectable effect on its own, but that BMP seemed to partially limit the EGF-induced proliferation (Figure 5B). To quantify and extend these observations, we performed immunofluorescence for Ki67, Olig2, the neuroblast marker doublecortin (DCX), and the mTORC1 readout pS6 (Figure 5C). Quantifications confirmed that EGF, but not EGF+BMP, stimulated a statistically significant increase in Ki67+ proliferating cells relative to Vehicle (Figure 5D). EGF is reported to increase the number of Olig2+ TAPs (Lindberg et al., 2012); we found that the EGF and EGF+BMP groups both exhibited statistically significant increases in Olig2+ cells relative to Vehicle, but that the EGF+BMP group was significantly lower than the EGF group (Figure 5E). Likewise, pS6 immunoreactivity is mainly expressed in TAPs, and was significantly increased in the EGF but not EGF+BMP groups relative to Vehicle (Figure 5F). Neuroblast numbers were decreased to a similar extent in both the EGF and EGF+BMP groups relative to Vehicle (Figure 5G). Given the massive proliferative response to EGF, it was not possible to reliably quantify numbers of GFAP+ astrocytes or ventricle-contacting GFAP+Ki67+ NSCs in this paradigm. Notably, while infusion of BMP by itself showed a tendency to decrease Ki67 and DCX levels compared to Vehicle infusion, it did not induce statistically significant changes for any of the markers examined.

Together, these quantifications reveal that in an *in vivo* context, TAP proliferation is induced by exogenous EGF when EGF is administered alone but not when it is co-administered with BMP.

### Inhibition of endogenous BMP signaling promotes NSC activation *in vivo*

**Figure 6:**
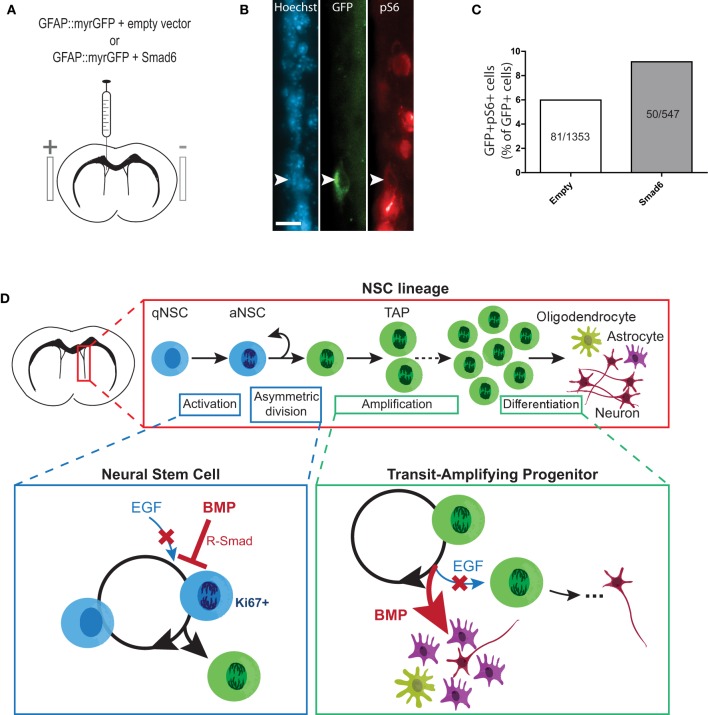
**Inhibition of BMP-SMAD signaling in NSCs promotes mTORC1 activity *in vivo*. (A–C)** *In vivo* electroporation of SMAD6 upregulates mTORC1 signaling in quiescent GFAP-positive NSCs. Experimental paradigm of *in vivo* adult brain electroporation (see Materials and Methods for details) **(A)**. Representative immunofluorescence images of GFAP::myrGFP-expressing NSCs that were double-labeled with pS6 **(B)**. Counts of GFAP::myrGFP cells that were double-labeled with pS6 (data pooled from *N* = 7−8 per group) **(C)**. **(D)** Summary figure: EGF-induced proliferative expansion of TAPs is dominantly suppressed by BMP via distinct stage-specific mechanisms. (Top) The majority of NSCs in the SVZ niche are quiescent. Upon EGF receptor (EGFR) activation, an NSC divides asymmetrically to generate a TAP, which undergoes several rounds of proliferative expansion before generating differentiated neural progeny (under normal conditions, predominantly neurons). (Lower left) At the NSC stage, ligand-induced stimulation of EGFR promotes NSC activation to generate a TAP via asymmetric division. BMP-induced activation of receptor-regulated SMAD (R-SMAD) dominantly inhibits EGFR-mediated NSC activation to promote NSC quiescence. (Lower right) At the TAP stage, EGFR stimulation promotes proliferative expansion of TAPs. BMP dominantly inhibits EGFR-mediated TAP proliferation and promotes astrocytic differentiation. Thus, increased BMP signaling in the SVZ leads depletes the TAP population by promoting quiescence of upstream NSCs and differentiation of TAPs. Scale bar **(B)** = 25 microns.

Since it was not possible to study growth factor-induced changes in NSC proliferation in the infusion paradigm, we turned to an *in vivo* adult brain electroporation strategy that would allow selective targeting of NSCs within their niche (Barnabé-Heider et al., 2008). To determine whether inhibition of endogenous BMP signaling can lead to NSC activation, plasmids expressing GFAP promoter-driven GFP (GFAP::myr-GFP, to label NSCs) were mixed with plasmids expressing either SMAD6 (a negative regulator of BMP-induced SMAD signaling) (Hata et al., 1998) or CMV6 (empty vector). Plasmid mixtures were injected into the lateral ventricles of adult mice and co-electroporated into the ventricle walls (Figures 6A,B). Immunofluorescence analysis of the SVZ 3 days post-electroporation revealed that in mice electroporated with empty vector, 5.98% (81 of 1353 cells) of GFAP::myr-GFP-expressing NSCs co-labeled with the mTORC1 readout pS6 (i.e., had undergone activation) (Figures 6B,C). In mice electroporated with SMAD6, this increased by 52.8%, to 9.14% of GFAP::myr-GFP-expressing NSCs (50 of 547 cells) (Figure 6C). Thus, endogenous BMP-SMAD signaling constitutively suppresses NSC activation, and acutely inhibiting endogenous BMP-SMAD signaling is sufficient to allow an increase in NSC activation.

## Discussion

The present study investigates signal integration during proliferative expansion of TAPs and their upstream activated NSCs. Strict regulation of this process is essential for maintaining a pool of TAPs that can participate in the ongoing replacement of specific neuronal and glial populations and that can dynamically modulate production of neural cells in response to changing local needs (as occurs following tissue injury). Here, we provide new insights into how TAPs and activated NSCs integrate two major pro- and anti-proliferation pathways, EGF-mTOR and BMP-SMAD (Figure 6D).

### Proliferative expansion of TAPs

EGF-induced mTOR signaling is emerging as a central regulator of adult neurogenesis. In our previous work, we demonstrated that EGF-induced mTORC1 signaling plays a critical role during proliferative expansion of the TAP population (Paliouras et al., 2012). mTORC1 activity was present in the majority of proliferating cells in neurosphere cultures *in vitro*, and in the SVZ was localized primarily to GFAP-negative cells that were positive for Ki67 and Mash1 (Paliouras et al., 2012). Such a phenotype identifies TAPs, but more recent studies suggest it may also include activated NSCs, which downregulate GFAP expression and upregulate Nestin (Codega et al., 2014).

We found here that, at the level of TAPs, BMP exerts completely opposing and highly dominant effects compared to EGF. A side-by-side comparison of multiple families of SVZ neurogenesis regulators confirmed that EGF is a particularly prominent pro-proliferative signal and demonstrated that BMP is a powerful anti-proliferative signal for neural precursors. BMP powerfully suppressed overall neural precursor proliferation in neurosphere-based assays, contrasting the effects of EGF, FGF, PDGF, VEGF, and SHH, other SVZ neurogenesis regulators. TAPs represent the vast majority of cells in neurospheres and neurosphere-derived adherent cultures. Dose-response experiments were performed to study how TAPs respond to simultaneous exposure to BMP and EGF, and showed that BMP's anti-proliferative effects were unchanged by increasing and excess levels of EGF, while conversely, EGF's pro-proliferative effects were suppressed in a dose-dependent manner by BMP. Notably, BMP-induced reductions in markers of cell proliferation (adherent cultures) and colony size (neurosphere cultures) were paralleled by powerful suppression of TAP markers such as Sox2, Olig2, and the mTOR pathway, and concomitant stimulation of differentiated neural markers (predominantly GFAP). *In vitro* analyses further revealed that BMP's anti-proliferative activity rapidly arrested EGF-induced cell division, stimulated differentiation, increased retention of cells in the G0-G1 phase of the cell cycle, and marginally increased cell death. These effects of BMP were opposite to those of EGF, and occurred regardless of coincident treatment with saturating concentrations of EGF, revealing that, *in vitro*, BMP is highly dominant to EGF on TAPs.

We hypothesized that BMP's anti-proliferative effect may operate via direct suppression of EGF-induced mTORC1 signaling, based on its dominant effects when co-administered with EGF, together with the identical kinetics of proliferation inhibition following BMP treatment and EGF withdrawal (CFSE experiment). However, unlike Rapamycin, which suppresses EGF-induced proliferation by directly blocking mTORC1 activity, BMP suppressed proliferation without decreasing mTORC1 activity. Thus, BMP treatment and EGF withdrawal suppress proliferation via distinct mechanisms. Inhibition of proliferation and expression of differentiation markers reached their maximum 1–2 days after the peak of BMP-induced SMAD signaling; this suggests that BMP initiates a differentiation process, but that the cells don't fully differentiate and stop proliferating until a few days later. Interestingly, proliferation recovery following transient Rapamycin treatment was paralleled by recovery of the 4EBP but not the S6 branch of mTORC1 signaling, consistent with a previous report that 4EBP rather than S6 kinase may be the main mTOR proliferation effector (Hartman et al., 2013).

We performed ICV growth factor infusions to validate these findings *in vivo*, finding both similarities and differences. When administered simultaneously *in vivo*, BMP was indeed found to attenuate EGF-induced expansion of markers of cell proliferation (Ki67) and TAPs (Olig2, pS6). However, there was an apparent difference in the effects of BMP on endogenous vs. EGF-induced proliferation. Specifically, BMP infusion by itself had no statistically significant effects on endogenous levels of Ki67 or TAPs, although a tendency for reduced Ki67 was noted. The most likely explanation for this difference is that endogenous ependymal-derived noggin may be capable of blocking exogenously administered BMP (Lim et al., 2000).

### Activation of NSCs

BMP was also found to have opposing and dominant anti-proliferative effects over EGF at the level of activated NSCs. Because NSCs represent only a few percent of cells within neurosphere-derived cultures or within the SVZ *in vivo*, measuring changes in NSC activity requires specific assays that can distinguish NSCs from their more abundant TAP progeny. *In vitro*, we used the neurosphere culture system to perform quantitative colony-forming assays. BMP treatment strongly inhibited EGF-induced neurosphere formation, and notably, this was not accompanied by an increase in under-sized colonies, suggesting that BMP had a quiescence-promoting effect on neurosphere-initiating NSCs. Consistent with this, self-renewal assays of BMP-treated neurospheres showed that they retained an almost normal complement of neurosphere-initiating cells, despite being reduced by more than 80% in neurosphere number and 65% in cells/neurosphere. Moreover, these BMP-treated NSCs generated significantly larger neurospheres when returned to a normal EGF-containing proliferation medium. These *in vitro* data indicate that BMP treatment does not cause a loss of NSCs (i.e., as would occur following terminal differentiation, death, or senescence); rather, BMP stimulates NSCs to enter a state of reversible quiescence that reduces their immediate TAP production but endows them with an enhanced capacity for subsequent proliferative expansion.

To test whether BMP constitutively restrains NSC activation *in vivo*, we turned to a recently developed *in vivo* adult brain electroporation protocol (Barnabé-Heider et al., 2008). Using this approach allowed us to identify and target ventricle-contacting GFAP-expressing NSCs for genetic manipulation. Previous studies suggest that 13.3% of NSCs express EGFR (Pastrana et al., 2009), representing NSCs that are either activated or primed for activation. Three days after electroporating quiescent GFAP+ NSCs with a control empty vector, we found that 6.0% expressed the mTORC1 readout pS6. Electroporating GFAP+ NSCs with a plasmid overexpressing SMAD6, a negative regulator of BMP-induced SMAD signaling, increased the percentage of electroporated NSCs that acquire mTORC1 activity to 9.1%, an increase of over 50%. It is not yet established whether this mTORC1 activity begins at the stage of stem cell activation (as occurs in muscle stem cells) (Rodgers et al., 2014) or upon generation of a TAP, however these data support a model in which BMP signaling constitutively represses NSC activation, and relieving this inhibition is sufficient to allow initiation of proliferative expansion. Interestingly, the question of whether prolonged suppression of BMP signaling would lead to exhaustion of the SVZ NSC pool has not yet been answered. In the dentate gyrus neurogenic niche, where NSCs have distinct growth and differentiation properties (Seaberg and van der Kooy, 2002), consensus has also not been reached on this question, with some studies reporting that that loss of BMP signaling leads to an eventual loss of NSC activity (Mira et al., 2010) and others reporting no such loss (Bonaguidi et al., 2008; Gobeske et al., 2009; Guo et al., 2011).

Together, the present data enable us to begin developing a picture of how pro- and anti-proliferative signals are integrated during proliferative expansion of the TAP pool (Figure 6D). Interestingly, a BMP signaling increase during aging may be a factor contributing to age-related decline in NSC activity (Yousef et al., 2015), suggesting that excessive quiescence signaling can perturb NSC functions under physiological conditions *in vivo*. Importantly, BMP-SMAD and EGF-mTOR represent only selected components of what is most likely a complex, multi-step and multi-factorial integration process. Indeed, while BMP suppression increased NSC activation, the majority of NSCs remained quiescent within the 3 day time-frame examined, suggesting that other players are involved (Ottone et al., 2014).

### Conflict of interest statement

The authors declare that the research was conducted in the absence of any commercial or financial relationships that could be construed as a potential conflict of interest.
